# Differential modulation of corticospinal excitability during haptic sensing of 2-D patterns vs. textures

**DOI:** 10.1186/1471-2202-11-149

**Published:** 2010-11-25

**Authors:** Sabah Master, François Tremblay

**Affiliations:** 1 School of Psychology, University of Ottawa, Ottawa, Ontario K1N 6N5, Canada; 2 School of Rehabilitation Sciences, University of Ottawa, Ottawa, Ontario K1H 8M5, Canada; 3 Élisabeth Bruyère Research Institute, Ottawa, Ontario K1N 5C8, Canada

## Abstract

**Background:**

Recently, we showed a selective enhancement in corticospinal excitability when participants actively discriminated raised 2-D symbols with the index finger. This extra-facilitation likely reflected activation in the premotor and dorsal prefrontal cortices modulating motor cortical activity during attention to haptic sensing. However, this parieto-frontal network appears to be finely modulated depending upon whether haptic sensing is directed towards material or geometric properties. To examine this issue, we contrasted changes in corticospinal excitability when young adults (n = 18) were engaged in either a roughness discrimination on two gratings with different spatial periods, or a 2-D pattern discrimination of the relative offset in the alignment of a row of small circles in the upward or downward direction.

**Results:**

A significant effect of task conditions was detected on motor evoked potential amplitudes, reflecting the observation that corticospinal facilitation was, on average, ~18% greater in the pattern discrimination than in the roughness discrimination.

**Conclusions:**

This differential modulation of corticospinal excitability during haptic sensing of 2-D patterns vs. roughness is consistent with the existence of preferred activation of a visuo-haptic cortical dorsal stream network including frontal motor areas during spatial vs. intensive processing of surface properties in the haptic system.

## Background

Haptic sensing involves bringing the hand in contact with objects and surfaces to determine their properties. In the case of surfaces, the presence of repeated raised elements characterizes textures, of which roughness is a major perceptual attribute. Recognition of 2-D patterns is another perceptual attribute provided by surfaces. Geometric properties such as the relative orientation of 2-D patterns are processed differently in the somatosensory system from material properties such as roughness and hardness. Neurons in Brodmann's area 1 have been shown to respond preferentially to surface features including roughness and texture [1,2], whereas neurons in Brodmann's area 2 are sensitive to differences in shape primitives, such as edges and curvature [2,3]. In primates, removal of areas 1 and 2 impairs performance on texture and shape discrimination tasks, repectively [2].

At the neural level, material properties are intensively coded, meaning that they can be distinguished based only on the amplitude of neural output at the cortical level [1,4-11]. In contrast, behavioural evidence has shown that geometric properties are likely spatially coded and must be mapped to a specific location within a particular frame of reference [12]. The haptic modality encodes geometric surface properties relatively inefficiently compared to material properties such as roughness [12]. This inefficiency can be attributed to several factors. Firstly, physical limitations of mechanoreceptor number and density lead to difficulties in the integration of limited inputs, as from a single 2-D raised element on a planar surface [12]. Secondly, the exploratory strategy for identifying a 2-D pattern is contour following, which entails a high working memory load [12-14]. Finally, spatial coding requires intensive coding to be completed first so that each 2-D raised element can first be detected, before it is located within the frame of reference, making it more computationally demanding than intensive coding alone [12].

Behavioural evidence has shown clear differences in the efficiency with which the haptic system processes intensively and spatially coded properties, observers showing faster responses with roughness discrimination than for identification of the relative orientation of 2-D raised elements, a key element involved in pattern discrimination [12]. Recent neuroimaging studies have contrasted the areas activated during texture and shape discrimination, revealing that different brain regions are preferentially activated during the haptic processing of material and geometric properties [15-19]. For instance, in participants making judgements about surface texture, more activation was seen in ventral and posterior regions including the parietal operculum and posterior insula along with activation in ventrally located extrastriate visual areas (e.g., lingual gyrus) [15,16]. Such activation pattern for haptic texture seems to reflect the preferential recruitment of a ventral stream specializing in object *perception *and semantic object representations [15,16]. In contrast, shape discrimination elicited preferential activation in frontal motor regions (e.g., premotor cortex) and in dorsal parietal areas commonly associated with reaching and grasping behaviours (e.g., intra-parietal sulcus, IPS; superior parietal gyrus, SPG); indicating a tendency for the recruitment of a dorsal stream for *action *when geometric properties about touched objects are processed [15-19]. Attending to geometric properties of objects either in the tactile or visual modality also leads to robust activation in the region of the lateral occipital complex (LOC), an area which seems critical for object recognition [15-19]. It is important to stress that the reported selectivity of activation for either texture (directed ventrally) or shape (directed dorsally) is more relative than absolute; the two forms of haptic processing relying to a large extent on an overlapping cortical network [15,16].

In recent investigations, we used transcranial magnetic stimulation (TMS) to examine task-dependant modulation in corticospinal excitability when participants were engaged in haptic sensing [20-23]. These investigations revealed that activation of a haptic network for discrimination of 2-D raised symbols resulted in enhanced excitability of the motor cortex, compared to performance of the same movement without the sensing component, or when attention was distracted away from the task [21]. This attention-related modulation of corticospinal facilitation during haptic sensing suggested that the observed effects were due to top-down mechanisms, including activation of a haptic sensing network involving somatosensory, multisensory, and motor areas [24]. However, this previous work investigated only modulation in the context of haptic sensing of the geometric properties of a surface, i.e. discrimination of the relative orientation of 2-D symbols. Given the reported differences in terms of complexity of neural processing and distribution of cortical activation between texture and shape discrimination by touch, we sought to determine in the present report whether such differences would lead to an increase in corticospinal excitability when participants are engaged in task conditions involving spatial coding (i.e. sensing the orientation of a 2-D pattern) versus intensive coding (i.e., sensing differences in degree of roughness) of surface features.

## Results

### Task performance

**Figure 1:**
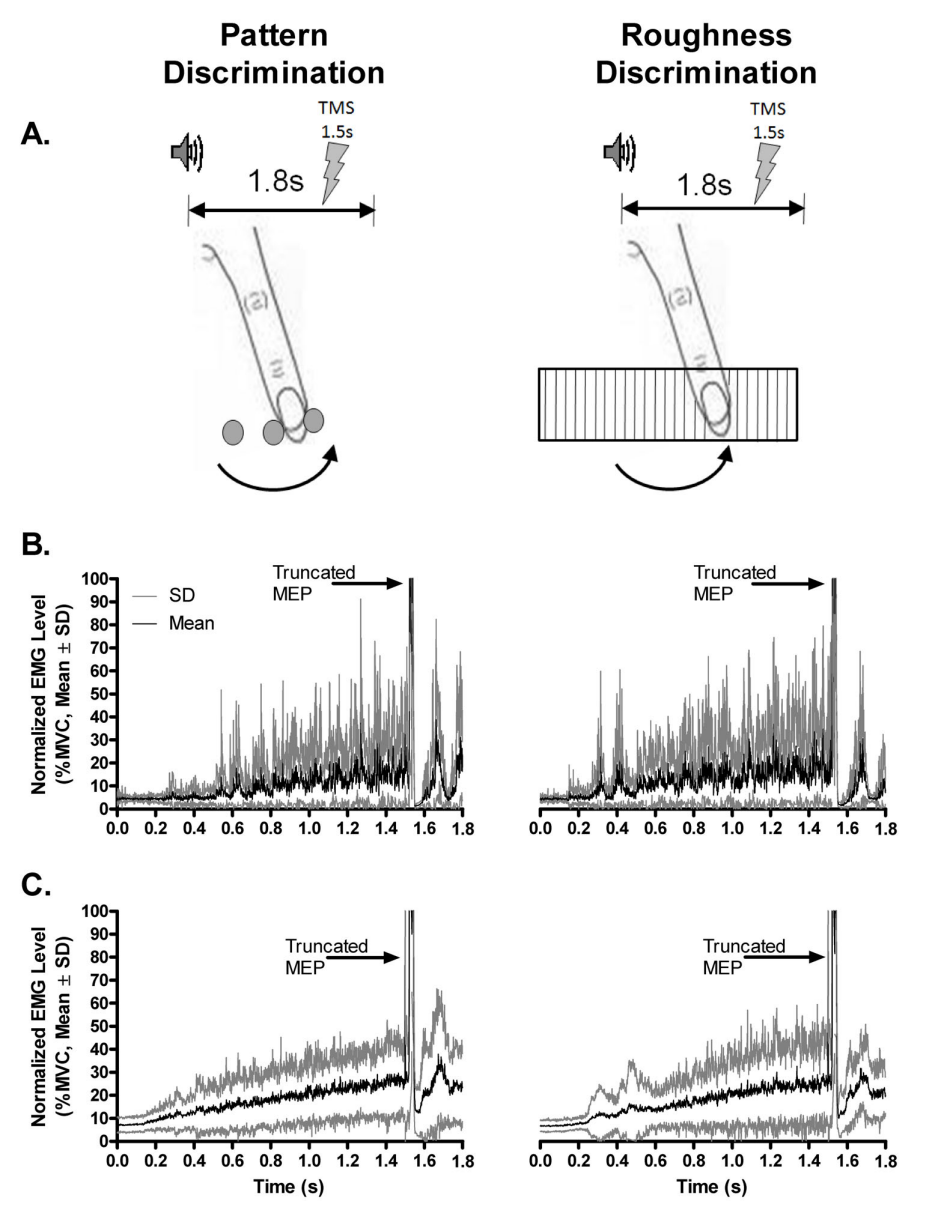
**Task paradigm used to assess corticospinal excitability**. A. In both discrimination tasks, participants were trained to produce a single stroking index finger movement in sync with the sound of a tone lasting for 1.8 s. In the pattern task, the finger moved over three circular stickers placed in a row, with the last circle off-set either upwards or downwards relative to the first two (3.2 mm radius, 10 mm centre-to-centre). Participants were asked to report whether the last circle was up or down. The roughness task required a judgement about the relative roughness of two grating surfaces, whose spacing between elements differed by 25%. Participants used the same stroking action to determine whether the surface presented was either the rougher (1 mm grating) or smoother (0.75 mm grating) of the two. In each trial, the TMS pulse was set to trigger 1.5s after the tone, corresponding to the time point when the finger was moving towards full abduction. B. Individual example of typical muscle activation patterns elicited in the FDI during execution of the index finger stroking action in the two tasks (right-handed male, aged 23 years). The traces represent the mean with associated SD of the rectified electromyographic (EMG) activity normalized as a percentage of the participant's maximal rectified average EMG value for all 16 trials under each task condition. C. Similar representation as in B showing the overall task-related pattern of EMG activation in all subjects (n = 18). The trace represents the mean (± SD) of all participants' normalized rectified average EMG activity level. Note the close similarity in the pattern of muscular activation between the two tasks. The amplitude of MEP's is truncated in both B and C because the scale has been adjusted to show the level of background EMG activity.

In general, participants exhibited very reliable discrimination performance in the two tasks, although they tended to be better at discriminating patterns as compared to surface roughness (84.4 ± 11.4% vs. 93.9 ± 6.4%, respectively). While task conditions had a significant effect on performance levels (F_1,16 _= 13.21, p = 0.002), this actually reflected only a minor difference in terms of the number of errors (n_incorrect_/16 trials) between the two tasks (i.e., pattern, n_incorrect _= 1.0 ± 1.0 trials, roughness, n_incorrect _= 2.5 ± 1.8 trials). Gender had no effect (F_1,16 _= 0.72, p = 0.41) on performance levels. In terms of muscle activation, execution of the stroking action in the two tasks elicited a very similar pattern of activity in the FDI msucle, characterized by a sustained increase in activity as the index finger moved over either the pattern or grating presented (Figure 1B and 1C). As shown in Figure 1C, the average level of activation elicited in the period preceding the TMS pulse corresponded to ~ 25% of the MVC in the two task conditions (mean, 23.81% vs. 22.55%, pattern and roughness, respectively). Paired comparison revealed no difference in the normalized level of EMG activity between the two tasks in the 500 ms period preceding the TMS pulse (paired t-test, t_17 _= -0.09, p = 0.93).

### Task-specific corticospinal facilitation

**Figure 2:**
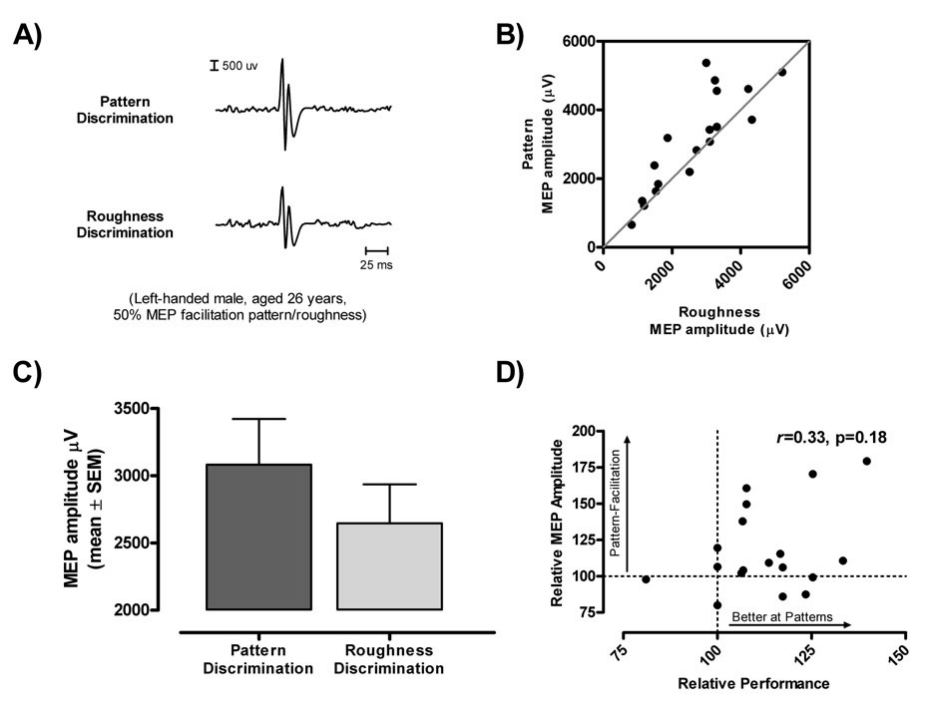
**MEP amplitudes during 2-D pattern vs. roughness discrimination**. A. Examples of task-related differential modulation in MEP amplitude under the two task conditions (left-handed male, aged 26 years, 50% MEP facilitation pattern/roughness). Each trace is an average of 16 responses. B. Scatter plot showing the relative distribution of MEP amplitudes in the two tasks for all participants. Note the tendency for MEP responses to be larger in the pattern discrimination task. C. Comparison of mean MEP amplitudes in the two task conditions (pattern and roughness discrimination). Each bar represents the mean of individual values computed under each task condition for all participants (n = 18). Note that the main effect of task condition was significant for the overall variations in MEP amplitude (p < 0.05). D. Scatter plot showing the distribution of individual MEP amplitude task ratios (% MEP_pattern_/MEP_roughnes_) against corresponding performance task ratios. Note that the two variables are not significantly related to one another.

The majority of the participants (11/18) exhibited larger MEP responses in the pattern, as opposed to, the roughness discrimination task. An individual example of such differential MEP modulation in the two task conditions is shown in Figure 2A. The relative effect of task conditions on MEP amplitude can also be appreciated in Figure 2B, where the individual MEP values measured under the two tasks have been plotted against each other. The differential effect of task conditions on MEP amplitude was confirmed by the ANOVA (F_1,16 _= 5.60, p = 0.03) owing to the larger MEP responses seen for the pattern task (18% increase, on average). Gender had no influence on MEP amplitude (F_1,16 _= 0.28, p = 0.60) and showed no interactions with task conditions (F_1,16 _= 0.13, p = 0.73). Variations in MEP latency (19.3 ± 1.8 vs. 19.1 ± 1.6 ms, pattern and roughness, respectively) and in the silent period (SP) duration (99 ± 37 vs. 94 ± 38 ms, pattern and roughness, respectively) were not influenced by task conditions (latency, F_1,16 _= 0.44, p = 0.51; silent period, F_1,16 _= 2.10, p = 0.17).

### Influence of task performance

The observation that participants tended to perform better at discriminating relative orientation, as compared to roughness, prompted a secondary analysis to determine if this increased performance was associated with the greater MEP facilitation during pattern discrimination. For this analysis, task ratios for MEP amplitude and performance (n_correct_/16) were computed as a percent of pattern over roughness (e.g., % MEP_pattern_/MEP_roughness_) for each participant, so that 100% would correspond to equivalence between the two tasks. We then used Pearson's *r *moment correlation to test for the presence of an association between MEP amplitude and performance task ratios. As shown in Figure 2D, this analysis failed to detect any significant association (*r *= 0.33, p = 0.18) between task-related MEP facilitation and performance.

## Discussion

In the present study, we examined task dependant corticomotor facilitation while participants were engaged in two different types of surface discrimination using active index finger movements, i.e. detection of 2-D spatial orientation and roughness discrimination. Our results reveal that for most participants pattern discrimination increases task-related MEP facilitation in the FDI, relative to roughness discrimination. In the following discussion, we will address the possible factors that may have contributed to this observed differential modulation of tactile-related motor facilitation by first considering task demands and bottom-up activity from peripheral afferents, and then top-down effects at the level of central processing.

### Task demands

As in our previous study showing tactile-related MEP facilitation, the movement conditions and level of background EMG were highly consistent between tasks, reflecting the fact that participants were able to reproduce the movement pattern at the prescribed speed from trial to trial. Thus the observed differences in motor cortical excitability could not have been due to the use of different motor exploration strategies during pattern and roughness discrimination. Given that participants used identical controlled timed lateral scanning movements for both tasks, differences in proprioceptive and kinematic factors could not have accounted for the observed enhancement in motor facilitation during the pattern discrimination, relative to the roughness discrimination [9,10]. The two tasks were also relatively easy to perform (accuracy > 80%), although participants did perform better on the pattern discrimination than on the roughness discrimination. However, as stressed earlier, this difference in performance represented only a minor difference in number of errors, given the limited number of trials tested in each task (n = 16). In this regard, this difference in performance can be considered as trivial. In line with this, secondary analysis failed to reveal any association between performance and MEP facilitation (Figure 2D).

### Contribution of bottom-up activity from peripheral afferents

The major difference between the two task conditions in terms of afferent input was the nature of the cutaneous feedback. The Merkel discs and associated slowly adapting type I (SAI) afferent system would have been most important in providing the coding information for the type of roughness discrimination used in the present study (spacing between raised elements > 0.2 mm), although the rapidly adapting type I (RAI) afferent system may also have contributed [11,25,26]. At the level of S1, inputs from SAI afferents are processed by cortical neurons that fire at higher rates for coarser textures, and the output of these neurons is thought to give rise to the percept of roughness [8,11,12,25,27]. Indeed, roughness has been shown to be coded intensively at the cortical level by neurons recorded in macaque monkey areas S1 and the secondary somatosensory cortex (S2), with a majority of these graded-response cells showing increased firing in response to rougher textured surfaces with larger groove widths [1,4-11]. Further, this activity has been shown to be minimally influenced by the difficulty of the texture discrimination (the percent difference in the spacing of raised elements), with such effects being minor relative to the observed changes in somatosensory cortical firing rate correlated with roughness [9,10].

Psychophysical data on response times and accuracy also suggest that roughness is coded intensively in the somatosensory cortex, while geometric properties such as relative orientation and position are likely resolved by comparing spatially distributed deformation patterns [12]. This has been shown to be a less efficient process due to the nesting of intensive coding within this computation, and the requirement for a spatial reference frame [12]. However, due to the intensive nature of the roughness code, both textures would likely have elicited more neural activity in S1 and S2, roughness-selective cells firing at higher frequencies relative to the spatially selective firing pattern indicative of the spatial location of a stimulus during pattern discrimination. For the pattern discrimination used in the present study, there would also have been less activity at the level of inputs from SAI afferents, since these afferents would have responded only to the edges in the pattern, which contained far fewer edges than the regularly ridged textures. The roughness discrimination, on the other hand, would have elicited much SA1 afferent activity given the spacing between the raised elements of the gratings used in the present study. RAI and PC afferent activity would also have been much lower during the pattern discrimination, due to the very low frequency of vibrations generated at the fingertip in response to the 2-D contours relative to those generated by the movement of the fingertip across the two textures [11,26-28]. The roughness discrimination thus also likely resulted in more peripheral afferent tactile activity than the geometric pattern discrimination. Whereas the relative roughness of a texture could in theory be extracted centrally as early as area 3b in S1, shape discrimination would likely require further processing in area 1 where speed of finger movement could be integrated with spatially distinct firing patterns to gain an overall representation of the pattern being scanned [29]. However, further processing in S2 or area 2 would likely not have been necessary for the simple 2-D pattern discrimination used in the present study [29]. The roughness discrimination would therefore have resulted in more somatosensory cortical activity overall, although this processing would have been simpler and more efficient than that required for the pattern discrimination.

It is possible that the intensity and diversity of peripheral afferent inputs, and the resulting activity in S1 and S2, could have determined motor cortical excitability during active tactile discrimination. The dense anatomical and functional connectivity between these regions and the primary motor cortex (M1) provides support for the presence of such bottom-up effects [30,31], and leads to the prediction of increased motor facilitation during the exploration of the densely packed raised elements composing the two textures as compared to during exploration of the relatively sparse elements (three circles) composing the two patterns. Indeed, task-specific increases in cutaneous afferent discharge have been shown to transiently increase both spinal and motor cortical excitability [32]. However, in the present study, only a small minority of individuals (3/18) showed evidence of greater MEP facilitation during active stroking of textured surfaces as compared to patterns. Therefore, simple bottom-up mechanisms associated with the degree and quality of tactile afferent feedback reaching M1 cannot account for the observed enhancement in task-related motor facilitation during the pattern discrimination.

### Contribution of top-down central factors

The present findings seem compatible with a top-down central origin for the observed difference in motor facilitation. This explanation is in line with our previous findings on the importance of attention to discrimination for tactile-related MEP facilitation. In this regard, the possibility that lower attention levels could have contributed to the decreased MEP facilitation associated with the roughness task seems highly unlikely for the reasons given above concerning the trivial nature of the observed differences in performance between tasks and the absence of any relationship between performance and MEP amplitude. Instead of difference in attention level, the explanation may lie with neuroimaging observations of a relative increase in the activation of a dorsal processing stream for *action *during the haptic processing of geometric properties such as 2-D patterns, as compared to during processing of material properties such as textures [15,21,25,33]. However, it is important to note that both of these types of stimuli would activate both somatosensory processing streams, but to varying degrees [15]. For example, texture is also important for determining how tightly to grasp an object, providing information regarding the amount of friction between the skin and the surface [11]. Nevertheless, texture may be less important in providing information related to grasping and manipulating objects compared to object shape or 2-D patterns, which provide inputs that help to determine how the fingers and hand are shaped [15].

Neuroimaging evidence also indicates that primary visual and dorsal extra-striate multisensory areas, such as the LOC, are also selectively activated during haptic shape recognition and imagery, compared to texture discrimination and imagery [15-19]. Haptic texture discrimination and imagery, on the other hand, preferentially activate the ventrally located medial occipital complex (MOC) and the inferior extra-striate regions [16,19]. These activations reflected both bottom-up somatosensory inputs and top-down modulation from the posterior parietal cortex [15,24]. Furthermore, during haptic shape discrimination in particular, a selective network was revealed involving bottom-up inputs from the S1 to the IPS and LOC, and top-down modulation from these regions to S1 [34]. Comparing shape to texture processing in the visual modality also revealed similar patterns of differential brain activation during these two types tasks [35]. Indeed, recent evidence suggests that both visual and haptic peripheral inputs lead to the activation of the same central networks for texture and shape processing [36,37]. Thus, segregation between the processing of texture and shape in the haptic and visual modalities may reflect common ventral and dorsal visuo-haptic networks specializing in the processing of texture and shape, respectively [15].

On the other hand, it has also been argued that spatial properties and shape are not processed by the dorsal parietal areas thought to make up the somatosensory dorsal stream for action (*where/how*) at all, and that evidence for shape processing in parts of the IPS may indicate that some parts of this region may contribute to the ventral, and others to the dorsal, somatosensory processing streams [29]. However, it is important to note that this explanation is based on studies using familiar shapes and objects that would likely preferentially activate ventral areas given their emotional significance and ability to trigger past memories. In the present study, we were interested only in motor cortical excitability during haptic identification of simple 2-D patterns, without much contribution of these other emotional and long-term memory factors. Although shape and texture could both be used as inputs to both dorsal and ventral stream networks [38], the present findings indicate that pattern discrimination may increase activation of motor areas to a greater degree relative to roughness discrimination. However, it is important to note that the haptic system is generally more tuned to 3D object perception than to 2-D pattern discrimination, such that the ability to recognize real objects is much better than recognition of raised line drawings [13,39,40]. This can be explained in part by the fact that a broader range of exploratory procedures is available for 3D objects, which changes the quality and quantity of information obtainable during 3D haptic sensing. Thus a stronger dorsal involvement may be expected when observers discriminate 3D objects as compared to simple 2D lined patterns, and this comparison would be an interesting avenue for future study.

These findings might also be interpreted in the context of the requirement for increased mental resources for the pattern discrimination due to the less efficient nature of spatial processing in the cortex, relative to the intensive processing presumably used during the roughness discrimination [12,41]. It is possible that the type of spatial haptic processing required by the pattern discrimination could have primed the motor cortex for action more than the intensive type of processing likely used for the roughness discrimination [15]. Both the relative functional importance of the type of afferent input (for example for motor activities such as shaping the fingers for grasping) and the complexity associated with processing the stimuli at the cortical level likely contributed to the observed corticomotor facilitation during pattern discrimination.

## Conclusions

This work builds on our previous findings of enhanced motor cortical excitability during active tactile discrimination of 2-D symbols versus finger movements alone [21], highlighting the importance of the specific haptic sensing demands with regards to the type of stimuli (pattern discrimination vs. roughness discrimination) for corticomotor facilitation. Together with this previous work, the present findings may help in developing functionally relevant tactile discrimination tasks for the re-education of hand function, tasks which would recruit frontal, parietal and occipital multisensory brain regions preferentially to facilitate finger movements. Indeed, the evidence presented here merits further investigation, in particular with regards to how task difficulty could interact with these task-type effects on M1 [15].

## Methods

The Institutional Review Ethics Board approved the study procedure in accordance with the principles of the Declaration of Helsinki and informed consent was obtained before the experimental session. All assessments were performed in a controlled laboratory environment. Each participant received an honorarium for his or her participation.

### Participants

Twenty healthy young adults (10 males, 10 females, mean age ± SD, 22 ± 2.3 years) from the Ottawa area were included in the study. The majority of subjects were right-handed (16/20) according to the Edinburgh Handedness Questionnaire. Prior to the experimental session, all participants completed a medical questionnaire to ensure that there were no contra-indications to TMS and no antecedents of conditions likely to affect their performance in the tests. In addition, all participants were screened for the presence of undiagnosed peripheral neuropathies using a graduated Rydel-Seiffer tuning fork, which has been shown to be a valid and reliable instrument for assessing sensory nerve function in the extremities [42,43].

### Training in tactile discrimination tasks

Participants were trained to perform two tactile discrimination tasks involving active movement of the right index finger over a surface. The training first focused on the production of consistent stroking actions with the index finger from right to left in sync with a tone lasting 1.8 s. Then participants were trained in the two discrimination tasks, which consisted of two-alternatives forced choice tasks. As illustrated in Figure 1, in one condition, the task consisted of discriminating between two patterns formed by three circular stickers (3.2 mm radius, 10 mm centre-to-centre) disposed on a small wooden block (13 cm × 8.5 cm). Participants were required to actively stroke the pattern in sync with the tone and to report the orientation of the last circle as being either in the upward or downward direction relative to the two other preceding circles. In the second task, participants performed a similar stroking action with the index finger but this time the task consisted of making a judgement about the relative roughness of gratings. Specifically, participants were required to discriminate between two fine grating surfaces (2 cm X 9 cm), each pasted on a wooden block. One grating was perceived as rougher than the other one, owing to a 25% increase in spatial period (1 mm vs. 0.75 mm, 0.2 mm constant ridge width). As for the pattern task, participants actively stroked the grating in sync with the tone to determine whether the surface was either the smoother (0.75 mm grating) or rougher (1 mm grating) of the two. In both tasks, participants received appropriate training prior to testing to achieve stable levels of performance and reliable discrimination (i.e., ≥75% correct). During both training and testing, participants were asked to verbally report their judgement about either the pattern or the roughness presented, immediately at the end of the trial, and were rapidly prompted to respond if no report had been made after the trial completion.

### EMG recording and TMS

The recording techniques and TMS procedure have been reported previously (see [21]). Briefly, EMG activity was recorded using small auto-adhesive surface electrodes (10 mm diameter, Ag-AgCl) placed over the FDI of the right hand. EMG signals were amplified (100-500 μV/div), filtered (bandwidth, 16 Hz to 1 kHz), and digitized at 1 kHz (RMP-6004, Nihon-Kohden Corp.; BNC-2090, National Instrument Corp.). Magnetic stimulation was delivered with a Magstim 200 (Magstim Co. Dyfed, UK) connected to a figure-eight coil (70 mm loop diameter). To determine the optimal site to evoke MEPs in the contralateral hand muscles, the approximate location of the hand motor area on the left hemisphere was explored in 1 cm steps until reliable MEPs could be evoked in the target muscle (FDI). Following this procedure, the relaxed motor threshold was determined using the method advocated by Mills and Nithi [44]. Starting from supra-threshold intensity, the stimulator's output was gradually decreased in 1% steps until no MEP could be evoked for 10 consecutive stimuli. This TMS intensity corresponded to the lower threshold value.

From this point, the intensity was gradually increased until MEP's of at least 50 μV peak-to-peak amplitude could be evoked by ten consecutive stimuli. This latter intensity was recorded as the upper threshold value. The relaxed motor threshold was defined for each participant as the median intensity between the upper and lower threshold values. The TMS intensity was then fixed at 1.1 X threshold for the remainder of the experiment.

### Recording of EMG during maximal voluntary contraction (MVC)

The procedure for EMG recording during the MVC is described in Master and Tremblay [21]. Briefly, maximal EMG values were measured for the FDI muscle at the beginning of the testing session. Participants were asked to push their index finger as hard as they could against the resistance provided by one of the experimenters for the duration of a tone lasting 3000 ms. The procedure was then repeated to get three MVC recordings.

### Recording of MEP's during haptic sensing

Corticospinal excitability was tested under the two task conditions (pattern and roughness discrimination) with participants comfortably seated in a recording chair and blindfolded. The order of testing with the two tasks alternated between participants to control for potential confounders due to variations in attention level, motivation and fatigue [21,23]. In each task condition, trials were presented in a random order (n = 16) with the two alternatives for each task (ie., pattern discrimination: up/down; roughness discrimination: rough/smooth) being equally probable. For both tasks (Figure 1), TMS was set to trigger towards the end of the stroking finger movement at 1.5s in the course of the 1.8 s trial. Sixteen trials of 1800 ms epochs were recorded under each task condition.

### Rationale for task design and selection of tactile stimuli

Tactile stimuli were specifically chosen to control for the degree of difficulty between tasks so that task performance would allow sufficient time to investigate task-specific facilitation in response to TMS. For the pattern discrimination, the three circular stickers were spaced to span a natural index finger adduction movement during tactile exploration of surface features. Pilot testing indicated that movement duration of ~2 s was consistent with such a natural finger exploration. The spacing and offset of the last of the three circles for the pattern discrimination was determined by further pilot testing, asking participants if they were still sensing and making their decision regarding the displacement of the last circle upwards or downwards when TMS was delivered towards the end of the stroking movement. Similarly, the two textures for the roughness discrimination were selected on the basis of previous experiments indicating that discrimination of smooth gratings (≤ 1 mm spatial period, ridge-to-ridge distance) is more difficult than coarser gratings (1-3 mm, spatial period) [9]. The two grating surfaces therefore consisted of relatively smooth gratings whose spatial period differed by 25% (i.e., 0.75 vs. 1 mm). As shown by Sinclair & Burton [9], such a difference in spatial period between gratings is relatively hard to detect by observers when allowed only one single stroke per trial. Pilot testing confirmed that participants were still sensing the texture (as judged by verbal reports) when TMS was delivered towards the end of the movement. Thus, the 1.5 s delay chosen for the time of TMS delivery provided an optimal time point to examine MEP facilitation, as participants were actively engaged in haptic sensing for surface features (either spatial orientation or roughness).

### Analysis of MEP data and EMG traces

Although a total of 20 participants were initally included in the analysis, careful re-examination of individual recordings revealed technical errors in the settings (trial duration was not properly adjusted) that invalidated the data for two participants (one male, one female). Therefore the MEP and EMG analysis was performed on the remaining 18 participants (9 males, 9 females, 4 left handed). The details for the procedure for analysis of MEP data are given in Master & Tremblay [21]. Briefly, MEP amplitude (peak to peak), latency, and EMG traces were measured off-line and averaged to derive mean individual values. For the MVC, the EMG signals produced during the last 2000 ms epoch were rectified and averaged to get a maximal rectified average EMG value. For the background EMG activity during test trials, the signal in the 500 ms window preceding the TMS pulse was rectified on a trial-by-trial basis for each participant, and these rectified values were then averaged and expressed as a percentage of each particiant's maximal rectified average EMG value (see Figure 1B and 1C). Finally, the SP was estimated as the interval from MEP onset to the first sign of EMG return.

### Statistical methods

A paired-samples t-test was performed on EMG levels (expressed as a percent of each participant's MVC) recorded during the two tasks, pattern discrimination and roughness discrimination. MEP amplitudes were normally distributed (Shapiro-Wilk P > 0.05) and did not need to be transformed. Four repeated measures ANOVAs were used to determine the impact of task condition (pattern/roughness discrimination) and gender on each dependent variable: 1) discrimination performance,2) MEP amplitude, 3) MEP latency, and 4) SP duration. The significance level was set at P < 0.05 for all tests. All tests were performed using SPSS software version 17.0 for Windows^® ^(Chicago, IL, USA). Figures were prepared using GraphPad Prism version 5.02 for Windows (GraphPad Software, San Diego California USA, http://www.graphpad.com).

## Authors' contributions

SM participated in the design of the study, carried out the behavioral testing, performed the statistical analysis and drafted the manuscript. FT conceived of the study, and participated in its design and coordination and drafted the manuscript. Both authors read and approved the manuscript and its revisions.
